# Risk Factors and Seroprevalence of Hepatitis C among Patients Hospitalized at Mulago Hospital, Uganda

**DOI:** 10.1155/2011/598341

**Published:** 2011-08-09

**Authors:** J. I. O'Reilly, P. Ocama, C. K. Opio, A. Alfred, E. Paintsil, E. Seremba, A. N. Sofair

**Affiliations:** ^1^Yale University School of Medicine, New Haven, CT 06510, USA; ^2^Makerere University Medical School, Kampala, Uganda

## Abstract

The emergence of hepatitis C virus (HCV) and its associated sequelae in Africa is a cause for significant concern. Human immunodeficiency virus (HIV) positive patients are at an increased risk of contracting HCV infection due to similar risk factors and modes of transmission. We investigated the seroprevalence of hepatitis C in hospitalized HIV-positive and HIV-negative patients in Mulago Hospital, an academic hospital in Uganda. Blood samples were first tested for HCV antibodies, and positive tests were confirmed with HCV RNA PCR. We enrolled five hundred patients, half HIV-positive and half HIV negative. Overall, 13/500 patients (2.6%) tested positive for HCV antibodies. There was no difference in HCV antibody detection among HIV-positive and HIV-negative patients. Out of all risk factors examined, only an age greater than 50 years was associated with HCV infection. Traditional risk factors for concurrent HIV and HCV transmission, such as intravenous drug use, were exceedingly rare in Uganda. Only 3 of 13 patients with detectable HCV antibodies were confirmed by HCV RNA detection. This result concurs with recent studies noting poor performance of HCV antibody testing when using African sera. These tests should be validated in the local population before implementation.

## 1. Background

Hepatitis C virus (HCV) infection is a significant cause of morbidity and mortality worldwide; however, the prevalence and burden of HCV in sub-Saharan Africa have received little attention. World Health Organization data gathered from more than 130 countries estimated that more than 170 million people are currently infected with HCV, contributing to a worldwide prevalence of 3% [1]. The prevalence of HCV infection in the general population in sub-Saharan Africa is estimated at 3% to 5.3% [2].Despite this high prevalence, active surveillance for HCV is rarely performed. This is due partly to resource constraints, unreliability of available serological tests, the high cost of nucleic acid testing (PCR) and confirmatory testing, and unaffordable cost of treatment [3, 4].

Studies of HCV prevalence in Uganda have yielded inconsistent results [5]. The earliest study showed a 2.5% prevalence by antibody testing. Two studies of HCV in HIV-infected patients gave an HCV prevalence of 0.6% and 2.9% [6, 7]. However, none of these studies confirmed antibody positivity by testing for HCV RNA or a recombinant immunoblot assay (RIBA). A study of Ugandan blood donors reported an initial HCV antibody prevalence of 4.0%, but only 0.6% after RIBA confirmation [3].Another study among individuals with sickle cell disease found a 4% antibody prevalence in children and 8% in their mothers [8]. However, only 11% and 8% of the seropositive children and mothers, respectively, had confirmation by RT-PCR. There is also a significant difference in the sensitivities of available serological tests [9]. For example, in one study of prenatal screening of Ugandan women, there was 16.6% antibody prevalence identified during the initial screening [10]. Upon retesting with a different antibody test, less than half of these were positive. Most recently a study of hospitalized patients from Kampala showed discordant results between hepatitis C rapid assays, standard EIA testing, and the confirmatory HCV RNA testing [11]. Anti-HCV antibody testing was positive in 13% of patients by one or both of these initial tests. However, only 29% of these samples had detectable HCV RNA on confirmatory testing. 

In the developed world, hepatitis C is strongly associated with human immunodeficiency virus due to shared risk factors and modes of transmission [3, 12]. Much like HIV, HCV is transmitted through blood-to-blood contact. Intravenous drug use and the sharing of virus-contaminated needles constitute the primary risk factor for cotransmission in the developed world. In some communities of IV drug users, the rate of coinfection of HIV-infected users with HCV approaches 90% [14]. Other risk factors include accidental needle stick injuries, transfusion of contaminated blood and blood products, as well as nonsterile tattooing practices [15]. In addition, vertical transmission from mother-to-child occurs in both hepatitis C and HIV [16]. Sexual contact is another important mode of transmission for HIV in both the developing and the industrialized world [17]. Unlike HIV, sexually transmission of HCV has received mixed reviews in the literature [15]. However, it is plausible that certain sexual practices that may involve exposure to blood, such as unprotected anal sex, may increase the risk for HCV transmission [18]. Recent epidemiological studies have revealed that certain medical interventions, such as nonsterile needle injections of antiparasitic drugs in Africa during the colonial era (1920s–1950s), might have contributed to high prevalence of chronic blood-borne pathogens such as HCV [19, 20].

There is also evidence that concurrent HCV infection makes it more difficult for HIV-infected patients to tolerate highly active antiretroviral therapy (HAART), leading to worse outcomes [21–23]. HIV has been shown to accelerate the progression of HCV-related sequelae such as cirrhosis and hepatocellular carcinoma [24–26]. Given the recent successful implementation of highly active antiretroviral therapy in Uganda, patients with HIV can live significantly longer and have less chance of developing AIDS-related illnesses [27, 28]. In the absence of these typical opportunistic infections, chronic comorbid illnesses will become a greater cause for concern. Currently, HCV is generally not screened for in Uganda, even among the HIV-infected, due to monetary considerations. We therefore sought to investigate whether HIV-infected patients were more likely to have HCV infection and if the antibody testing was reliable enough to be used universally in Uganda. A positive correlation between HIV and HCV would suggest that HIV-infected patients be treated with a higher degree of suspicion for HCV infection. Our study compared cohorts of HIV-infected and HIV-non-infected patients for hepatitis C in an inpatient setting at an academic hospital in Uganda and to describe any risk factors associated with HCV infection in this cohort.

## 2. Methods

### 2.1. Study Participants

The study was conducted in Mulago hospital, a national referral hospital in Kampala, Uganda that admits over 12,000 patients a year, nearly half of whom are HIV positive. Patients 18 years old and above with a known HIV serostatus who were admitted to the medical wards were invited to participate in the study. HIV testing is normally performed on all willing patients of unknown serostatus in an HIV voluntary counseling and testing (VCT) program provided on all the wards of Mulago Hospital. Five hundred patients were randomly selected and consented to participate in this study. One half of the patients enrolled were HIV-seropositive, and the other half were HIV-seronegative. Translators were utilized for any patient unable to speak English.

After obtaining written informed consent, patients had approximately 10 mL of blood drawn for HCV testing. We also administered a questionnaire to all patients in order to define demographic, epidemiologic, and clinical variables. The study participants were not given any monetary incentives and those found to be HCV infected were not offered treatment as there are no national guidelines for HCV treatment. Patients testing positive for HCV infection, either by EIA or by PCR, were informed of their status and its ramifications by an experienced gastroenterologist and were invited to attend the gastroenterology clinic. They were also informed that the final tests to confirm infection would be delivered to them when the result became available. 

This study was approved by the Makerere University Faculty of Medicine Research and Ethics committee Review Board, the Uganda Council for Science and Technology, and the Yale University School of Medicine Human Investigation Committee.

### 2.2. Laboratory Analysis

Blood samples collected from study participants initially were tested for hepatitis C antibody using a Bioline rapid test kit (Bioline Medical Systems, Pasig City, Philippines). This rapid HCV antibody test was the preferred method of HCV detection in Mulago Hospital. The remaining blood was centrifuged within 4 hours of collection. The leukocyte band layer was extracted and added to 5 volumes of RNAlater solution in accordance with Ambion protocol (Austin, Texas, USA). RNAlater was used to preserve viral RNA during transport. The solution was stored at −20°C before transport to Yale University School of Medicine for further analysis. Per RNAlater protocol samples can be thawed during transit and refrozen without affecting the amount or integrity of recoverable RNA. 

RNA was extracted from the previously described samples using a Qiagen QIAamp Viral Kit (Valencia, California, USA). Extracted RNA was stored at −20°C until RT-PCR could be performed. Primers from the core region of the HCV genome were used to amplify the extracted RNA using a Qiagen one step RT-PCR kit as previously described [29]. PCR products were separated by agarose gel electrophoresis and visualized by ethidium bromide staining. Samples testing positive for HCV RNA by RT-PCR were confirmed by HCV sequence analysis. PCR products were purified using a Qiagen QIAquick PCR purification kit, and sequenced at the Keck Biotechnology Resource Laboratory at Yale University. Repeat HCV EIA testing was performed using an Ortho HCV version 3.0 ELISA test kit (Ortho-Clinical Diagnostics, New Jersey, USA).

### 2.3. Statistics

Data were analyzed using statistical analysis system (SAS) version 9.1 (SAS Institute Inc., Cary, North Carolina, USA). Demographic and epidemiologic characteristics of the study sample were described using descriptive statistics. For comparisons between HCV and HIV infection, as well as other variables, a two-sided *Z*-test to compare proportions was used at an *α* of 0.05.

## 3. Results

From January to February 2008, we enrolled 500 patients who attended the Mulago Hospital inpatient service, of whom 235/500 (47%) were male. The median age of the patients was 35 years. One half (250/50) of the patients were HIV positive. In the subgroup analysis shown in Table 1, patients aged 31–50 were significantly more likely to have HIV, and those over 50 years of age significantly less likely. The patients who identified themselves as farmers or unemployed were significantly more likely to have HIV infection; while those identified as students were significantly less likely to be HIV positive.

Overall 16 of the 500 (3.2%) patients tested positive for HCV antibodies using the Bioline rapid assay. Subsequently, all seropositive samples were retested with a confirmatory Ortho HCV 3.0 immunoassay. 13 of the 16 positive HCV antibody samples also tested positive by this immunoassay (2.6%). HIV status was not associated with HCV antibody detection in patients. There was no difference in HCV antibody detection among HIV negative (7 cases) and HIV positive (6 cases). Confirmation of HCV viremia was performed by the detection of HCV RNA using RT-PCR on all 13 HCV-positive antibody samples. HCV RNA was detected in only 3 of the 13 (23%) samples. Of these, 2 were detected in HIV-positive patients, and 1 was detected in HIV-negative patients. In addition, a cohort who tested negative for the presence of HCV antibodies were tested for the presence of HCV RNA. Interestingly, 5 of 89 samples randomly selected from the HCV antibody-negative HIV-positive patients tested positive for HCV RNA. All HCV antibody-negative HIV-negative patients tested negative for HCV RNA. 

As indicated in Table 2, quantitative analysis demonstrated that participants with ages greater than 50 were significantly associated with HCV antibody detection. Blood donors, patients receiving transfusions, patients with previous surgeries, and patients with tattoos or ritual scars were also more likely to have HCV. However, these other risk factors were not sufficiently powered to achieve statistical significance. 

Samples that had tested positive for HCV RNA were sent for RNA sequencing. These samples were matched against known databases for HCV. All amplifiable sequences were matched to genotype 1B.

## 4. Discussion

Our results indicate much variability between the screening and confirmatory testing for hepatitis C in Uganda. Screening for hepatitis C is performed by testing for antibodies in patients using commercially available assays. The confirmatory testing is PCR to detect HCV RNA viremia. Acute hepatitis C infection is generally thought to progress to chronic hepatitis C 75% of the time [30]. In this case, we would expect only 75% of antibody testing to be confirmed by PCR, since a quarter of patients will spontaneously clear the disease. However, in our study only 3 of 13 HCV-antibody positive samples, or 23%, subsequently tested positive for active HCV infection. As previous studies testing for hepatitis C infection in Uganda have shown, there have been marked discrepancies between the initial and confirmatory testing. Seremba et al. [11] were only able to confirm 29% of positive samples with RNA testing, and even showed marked discrepancies between different antibody tests. In our study, we initially tested for HCV using the Bioline rapid assay used in Mulago Hospital. Subsequently, 13 of 16 samples also tested positive with the Ortho third generation anti-HCV ELISA, which was chosen because it is a widely used immunoassay. Thus the Bioline rapid assay appears to be adequate in this population compared to other available immunoassays. However, antibody testing in Uganda may be a poor indicator of active HCV infection [4]. Studies comparing different antibody tests have noted poor performance when using sera from Africans using the Ortho anti-HCV 2nd and 3rd generation EIA test kits [3, 9]; these tests are normally very sensitive [31]. In this case, antibody tests developed based on sera from developed countries may not sufficiently capture the seroepidemiology of the disease. The hepatitis C epidemic is maturing in a subregion with a wide genetic diversity, so poor detection of this virus using narrow-spectrum tests may be expected. There remains the possibility that a larger percentage of Ugandan patients clear the disease; however, this seems unlikely to account for all of the noted discrepancy. 

Analysis of the demographics of HIV infection between our two cohorts reveals interesting findings. The HIV prevalence between the younger subgroups in each cohort was similar, but the middle-aged subgroup was significantly more likely to have contracted HIV. This correlates with an increased opportunity to acquire infection as one ages. The older subgroup was significantly less likely to have HIV, which may be due to decreased sexual risk factors, but is more likely due to the mortality associated with chronic HIV infection. Decreased prevalence of HIV infection was seen in students, a younger, more educated subgroup. In contrast, more HIV infection was seen in those less educated: farmers and the unemployed.

There are several limitations to our study and its results. Confirming our EIA results with RIBA testing may have given us a more accurate determination of antibody status. Since all samples must be transported overseas for PCR analysis, RNA degradation was possible despite precautions taken, although other studies have had success with this media [29]. Although the custom primer used during PCR had been validated internally at Yale, to our knowledge it has never been used on African sera. Finally, HCV RNA PCR is a technically demanding process, which may cause both false positives and false negatives to occur.

Given these constraints, it is informative to evaluate the results of antibody testing. The prevalence of hepatitis C infection detected by antibody testing was between 2.6% and 3.2% in this study, depending on the assay used. The lack of clear association between HCV and HIV infection is likely due to the fact that some risk factors commonly seen in the West, namely, injection drug use, are not common practice in most African countries. HIV currently is primarily transmitted by sexual contact in Uganda; the likelihood of HCV transmission though sexual contact is very low. The one risk factor shown in this study for HCV infection, an age over 50, is unlikely to be associated with HIV because elderly patients who contracted this HIV decades ago would have died by now since HAART was not readily available. Our results reinforce recent studies showing an association between blood borne infections and the use of contaminated medical instruments and unsterile practices in the past [19, 20]. These risks are primarily confined to older generations before safe practices were instituted. The data also suggest that risk factors for blood transmission, such as tattooing, transfusions, and surgery increase risk for HCV transmission. However, the study was not sufficiently powered to show statistical significance for these variables.

It is notable that 5 of 84 HIV-positive patients who initially tested negative for HCV antibodies were found to have HCV viremia. This phenomenon seems limited to HIV-positive patients, as none of the 25 HCV/HIV-negative patients tested had detectable HCV RNA. HIV-positive patients in Uganda are usually identified very late in the course of disease progression, with low CD4 counts and symptomatic AIDS-related illnesses. There is evidence to suggest that in patients with HIV-associated immune suppression the detection of HCV antibodies is more difficult [32–35]. 

In summary, the seroprevalence noted in this study appears to greatly overestimate the prevalence of HCV viremia in this cohort of Ugandan hospital patients. This discrepancy may be due to disease clearance or false positives in antibody testing, possibly related to chronic stimulation of the immune system from a variety of tropical infectious diseases. There remains an urgent need for these tests to be validated in each population prior to clinical utilization. An analysis determining if African sera tested for HCV antibodies can be accurately and cost-effectively confirmed by RNA PCR testing is also needed. Finally, it is currently unknown how the progression of HIV in Ugandan patients affects the detection of HCV antibodies in these patients. These issues would benefit greatly from a further study.

## Figures and Tables

**Table 1:** Demographic Characteristics of the Study Population.

Characteristic	HIV-Positive Cohort (*n* = 250)	HIV-Negative Cohort (*n* = 250)	*P* Value
Age			
18–30	38.4% (96)	33.6% (84)	0.303
31–50	56.0% (140)	38.0% (95)	<0.001
>50	5.6% (14)	28.4% (71)	<0.001
Gender			
Male	46.8% (117)	47.2% (118)	1.000
Female	53.2% (133)	52.8% (132)	1.000
Employment			
Teacher	3.6% (9)	2.4% (6)	0.603
Farmer	25.2% (63)	16.0% (40)	0.016
Fisherman	0.8% (2)	1.6% (4)	0.689
Shopkeeper	10.8% (27)	8.0% (20)	0.358
Driver	7.2% (18)	3.2% (8)	0.072
Student	1.2% (3)	10.4% (26)	<0.001
Community Worker	1.6% (4)	4.0% (10)	0.177
Unemployed	32.0% (80)	22.4% (56)	0.021
Other	26.8% (67)	25.6% (64)	0.839

**Table 2:** Risk factor analysis of study population.

Variable	HCV EIA positive	HCV EIA negative	Percentage	*P* value
HIV status				
Positive	6	244	2.4%	1.000
Negative	7	243	2.8%	
Age				
<50 years	7	95	6.8%	0.007
>50 years	6	392	1.5%	
Blood donor				
Yes	3	68	4.2%	0.600
No	10	418	2.3%	
Transfusion				
Yes	4	64	5.9%	0.160
No	9	421	2.0%	
Previous surgery				
Yes	3	45	6.3%	0.234
No	10	441	2.2%	
Tattoo or ritualized scaring				
Yes	2	15	11.8%	0.052
No	11	444	2.4%	
IV drug use				
Yes	0	3	0.0%	0.935
No	13	474	2.7%	
Sexually Active				
Yes	8	408	1.9%	0.078
No	5	78	6.0%	
Condom use (lifetime)				
Yes	1	16	5.9%	0.943
No	11	433	2.5%	
Sex for money (lifetime)				
Yes	1	27	3.6%	0.791
No	12	454	2.6%	
Sex while intoxicated (lifetime)				
Yes	4	67	5.6%	0.221
No	9	395	2.2%	
